# Differential diagnosis and long-term outcomes of non-atrophic duodenal changes in children

**DOI:** 10.3389/fped.2022.982623

**Published:** 2022-08-29

**Authors:** Sofia Kröger, Marleena Repo, Pauliina Hiltunen, Martine Vornanen, Heini Huhtala, Laura Kivelä, Kalle Kurppa

**Affiliations:** ^1^Department of Pediatrics, Tampere Center for Child, Adolescent and Maternal Health Research, Tampere University Hospital, Tampere University, Tampere, Finland; ^2^Celiac Disease Research Center, Tampere University, Tampere, Finland; ^3^Department of Pathology, Tampere University Hospital, Tampere, Finland; ^4^Faculty of Social Sciences, Tampere University, Tampere, Finland; ^5^Children’s Hospital and Pediatric Research Center, Helsinki University Hospital, University of Helsinki, Helsinki, Finland; ^6^Seinäjoki Central Hospital, University Consortium of Seinäjoki, Seinäjoki, Finland

**Keywords:** biopsy, duodenum, endoscopy, esophagogastroduodenoscopy, follow-up, gastroenterology, histology, pediatric

## Abstract

**Objectives and study:**

Gastrointestinal endoscopy is often performed when investigating abdominal complaints in children. While atrophic changes of the duodenal mucosa are usually caused by celiac disease, the prevalence and clinical significance of non-atrophic duodenal changes are less clear. We studied these issues in a large pediatric endoscopic cohort.

**Methods:**

Comprehensive data on clinical features, diagnostic findings and long-term outcomes of children who had undergone upper gastrointestinal endoscopy with systematic duodenal sampling were collected. Study variables were compared between children with non-atrophic changes and normal histology, and between those with non-atrophic changes who did and did not receive a diagnosis.

**Results:**

The study comprised 1,170 consecutive children, of whom 51 (4.4%) had non-atrophic and 315 (26.9%) atrophic duodenal changes and 804 (68.7%) normal histology. The most common non-atrophic findings were non-specific inflammation (*n* = 19) and intraepithelial lymphocytosis (*n* = 14). Patients with non-atrophic changes presented more often with blood in stools (23.5 vs. 11.3%; *p* = 0.009), anemia (43.2 vs. 36.5%; *p* = 0.028) and positive celiac serology (34.3 vs. 12.9%; *p* < 0.001) than those with a normal duodenum. Twenty-four (44%) of those with non-atrophic changes received an initial diagnosis, the most common of which were inflammatory bowel disease (IBD) (*n* = 8), *Helicobacter pylori* infection (*n* = 3) and food allergy (*n* = 3). The prevalence of the diagnoses did not differ from those with a normal duodenum. Those who received a diagnosis had more often blood in stools (37.5 vs. 11.1%; *p* = 0.027), anemia (70.6 vs. 20.0%; *p* = 0.002) and negative celiac serology (50.0 vs. 7.7%*; p* = 0.013) than those without diagnosis. During a follow-up of 6.1–13.3 years, five of the 12 initially undiagnosed seropositive patients developed celiac disease, and one patient also developed ulcerative colitis.

**Conclusion:**

Non-atrophic duodenal changes are relatively common and associated with anemia, blood in stools, and positive celiac disease serology. Excluding potential celiac disease, those without an initial diagnosis have a favorable long-term prognosis.

## Introduction

The differential diagnosis of pediatric gastrointestinal complaints can be challenging (1, 2). While laboratory testing and imaging may provide useful information, more invasive procedures, such as esophagogastroduodenoscopy (EGD) and colonoscopy are frequently needed to set the diagnosis (2, 3). Due to the weak association between endoscopic and microscopic findings (4, 5), mucosal biopsies are recommended to be taken even when there appear to be no visual abnormalities (3, 5, 6). While improving the diagnostic yield, frequent use of endoscopic studies with systematic sampling of the gastrointestinal tract has resulted in increased reporting of various histologic abnormalities of unclear significance. On the other hand, duodenal changes, in particular, may also be of major clinical significance as a normal duodenum is a prerequisite for the absorption of many essential nutrients.

By far the most common cause for advanced atrophic changes of the duodenal mucosa is celiac disease, the diagnosis of which is usually quite straightforward, at least when the characteristic intraepithelial lymphocytosis, villous atrophy, and crypt hyperplasia are present (7, 8). The more ambiguous inflammatory changes or other non-structural mucosal abnormalities have been associated, for example, with inflammatory bowel disease (IBD), certain viral and parasitic infections, and medications (7, 9–12), yet pediatric studies with systematic duodenal sampling reporting long-term outcomes are scant. The few papers published hitherto have focused mainly on intraepithelial lymphocytosis and celiac disease (4, 12–14), hence the need for additional studies with a broader view and longer follow-up time.

Our unit has a long tradition of using standardized procedures during pediatric gastrointestinal endoscopies (15) and maintaining systematic patient records. We utilized these benefits to study the prevalence and diagnostic outcomes of non-atrophic duodenal changes in children who had undergone EGD with systematic sampling.

## Materials and methods

### Patients, study design, and ethical considerations

**FIGURE 1:**
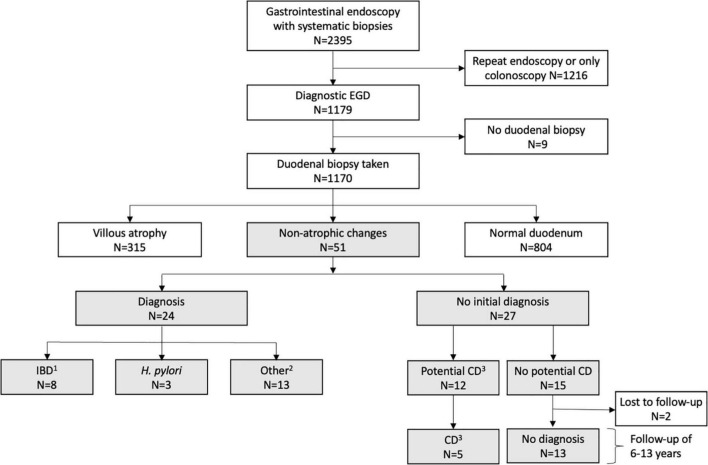
Flowchart of the study. ^1^Crohn’s disease (*n* = 6), inflammatory bowel disease unclassified (*n* = 2), ^2^gastroesophageal reflux disease (*n* = 4), wheat allergy (*n* = 1), cow’s milk allergy (*n* = 1), other food allergy (*n* = 1), mastocytosis (*n* = 2), postinfectious EBV colitis (*n* = 1), mevalonate kinase deficiency (*n* = 1), gastric ulcer (*n* = 1), polyposis (*n* = 1); ^3^elevated serum tissue transglutaminase antibody and/or endomysial antibody levels without diagnostic duodenal lesion. CD, celiac disease; EGD, esophagogastroduodenoscopy; IBD, inflammatory bowel disease.

The study was conducted at the Tampere Center for Child, Adolescent, and Maternal Health Research. The patient cohort was formed by collecting comprehensive medical data as of May 2020 of all children (<16 years of age at the time of endoscopy) who had undergone gastrointestinal endoscopy between 2007 and 2014 in the Department of Pediatric Gastroenterology (Figure 1). The endoscopies were limited to these years in order to obtain a minimum follow-up time of 6 years. Patients who had undergone only colonoscopies or had no duodenal sampling and those who had undergone repeated endoscopy were excluded. For the study comparisons, subjects were categorized on the basis of duodenal histology into those with normal findings and non-atrophic changes, and the latter group further to those who did and did not receive a diagnosis during the initial investigations (Figure 1). Those with atrophic mucosal changes, i.e., villous atrophy and crypt hyperplasia, were excluded from the comparisons. Non-atrophic changes were defined as any abnormal duodenal histological finding reported by the pathologist excluding the aforesaid atrophic changes.

The study follows the guidelines of the Declaration of Helsinki. The Department of Pediatrics, Tampere University Hospital granted the requested institutional approval for the collection of the patient data. According to the Finnish national guidelines, approval of the Ethical committee was not needed in this registry-based study. All data were analyzed anonymously, and the patients were not contacted at any stage of the study.

### Clinical data and laboratory parameters

Demographic data, clinical presentation, growth parameters, presence of previous chronic diseases, use of medications, and family history of gastrointestinal diseases were recorded from each study subject. In addition, blood hemoglobin (Hb; reference values from 94–130 g/L to 150–230 g/L based on sex and age), plasma albumin (from 28–44 g/L to 37–51 g/L), serum IgA class transglutaminase 2 (TGA; <7.0 U/L) and endomysial antibodies (EmA; titer 1: <5), erythrocyte sedimentation rate (ESR; <15 mm/h) and fecal calprotectin (<100 μg/g), were collected and categorized into normal or abnormal (16). All official diagnoses as set by the treating physician based on the diagnostic investigations either at the time of the initial endoscopies or during subsequent follow-up were also collected. Food allergies were diagnosed based on systematic elimination-challenge test, together with compatible histologic and laboratory findings. Hematochezia and melena were combined under a single entity of “bloody stools” signifying possible gastrointestinal bleeding.

### Endoscopies and histopathology

At least two mucosal forceps biopsy specimens from the lower esophagus, gastric corpus, and antrum and at least four samples from the duodenum were obtained from each patient during the whole study period. Since 2012, additional samples have been systematically taken from the duodenal bulb and middle part of the esophagus (17). The specimens were cut, stained, and evaluated by standard histopathology methods. Special stainings were applied as indicated. To avoid false negative diagnoses of celiac disease, particular attention was paid to the quality of samples and correct evaluation of the duodenal morphometry (18). The presence of non-atrophic duodenal changes such as granulomas, lymphangiectasia, increased number of specific inflammatory cells, and their significance were collected from pathology reports.

### Statistical issues

All statistical analyses were performed with SPSS version 25 (IBM Corporation, Armonk, NY, United States). Categorical values are expressed in percentages and quantitative variables as medians with upper and lower quartiles as these were found to be non-normally distributed by the Kolmogorov-Smirnov and Shapiro-Wilk tests. Chi-square test and Fisher’s exact test were used for comparisons of categorical variables and Mann-Whitney *U* test in quantitative variables. A *p*-value of < 0.05 was considered significant.

## Results

### Study cohort and diagnostic findings

The study cohort comprised 1,170 consecutive children who had undergone their first diagnostic EGD (Figure 1). Duodenal biopsies were taken from 99.2% of them and 4.4% had non-atrophic histologic changes, 26.9% atrophic changes, and 68.7% normal duodenal mucosa (Figure 1). Of children with non-atrophic changes, 47.1% received a diagnosis during the initial investigations, this figure being comparable to the figure for those with normal mucosa (35.9%, *p* = 0.110). The most common diagnoses in both groups were IBD (15.7 vs. 14.8%; *p* = 0.883), *Helicobacter pylori (H. pylori)* infection (5.9 vs. 3.1%; *p* = 0.281), food allergy (5.9 vs. 2.6%; *p* = 0.167), and mastocytosis (3.9 vs. 0.6%; *p* = 0.060). Of the patients who did not receive a diagnosis, 44.4% had elevated TGA and/or EmA without atrophic changes, i.e., potential celiac disease (Figure 1). Of all 1,170 study children, 20 had eosinophilic esophagitis. Six of these patients had atrophic duodenal changes, 14 normal duodenal histology and none non-atrophic mucosal changes.

### Clinical features associated with non-atrophic duodenal changes

Children with non-atrophic changes had more often blood in stools and anemia, family history of celiac disease, and positive celiac serology than did those with normal duodenal mucosa, whereas the groups did not differ in the other clinical and laboratory parameters (Table 1) or median levels of Hb (123 vs. 128 g/l, *p* = 0.069; data available from *n* = 599), ESR (8 vs. 8 mm/h, *p* = 0.575; *n* = 480), albumin (40 vs. 38 g/l, *p* = 0.229; *n* = 307) and calprotectin (192 vs. 67 μg/g, *p* = 0.447; *n* = 293). Prevalence of gastritis or histological abnormalities in the esophagus, terminal ileum, ascending colon, sigmoid colon, or rectum did not differ between the groups (data not shown).

**TABLE 1:** Comparison of clinical characteristics at the time of first esophagogastroduodenoscopy between children with non-atrophic duodenal changes and those with histologically normal duodenum.

	Non-atrophic changes, *n* = 51		Normal duodenum, *n* = 804		
		
	*n*	%	*n*	%	*P*-value
Girls	27	52.9	404	50.2	0.773
Symptoms					
* Abdominal pain*	26	51.0	494	61.4	0.138
* Diarrhea*	12	23.5	242	30.1	0.319
* Blood in stools*	12	23.5	91	11.3	**0.009**
* Heartburn*	12	23.5	221	27.5	0.538
* Poor growth or weight-loss*	10	19.6	131	16.3	0.536
* Constipation*	8	15.7	97	12.1	0.445
* Vomiting*	6	11.8	121	15.0	0.522
* Dysphagia*	2	3.9	35	4.4	1.000
Laboratory findings					
* Anemia^a^*	16	43.2	151	36.5	**0.028**
* Positive celiac disease serology*^b^**	12	34.3	65	12.9	**<0.001**
* Elevated ESR*^c^**	8	27.6	131	29.0	0.867
Previous chronic conditions					
* Asthma or allergy*	13	25.5	168	20.9	0.436
* Autoimmune disease*^d^**	4	7.8	37	4.6	0.299
Family history of gastrointestinal diseases					
* Celiac disease*	9	17.6	69	8.6	**0.041**
* Inflammatory bowel disease*	6	11.8	71	8.8	0.449
	**Median**	**Quartiles**	**Median**	**Quartiles**	
	
Age, years	8.5	3.3, 12.4	9.2	4.7, 13.3	0.157
Height, SD*^e^*	1.0	–0.2, 1.0	1.0	0.1, 1.0	0.723
Body mass index-SDS*^f^*	0.5	–0.8, 1.1	–0.1	–1.0, 0.7	0.074

Data were available for >90% patients except for ^a^70.9, ^b^62.9, ^c^56.1, ^e^69.1, and ^f^64.7%.

^d^Example, type 1 diabetes, autoimmune thyroid disease, rheumatoid arthritis, celiac disease; ESR, erythrocyte sedimentation rate; SD, standard deviation; SDS, standard deviation score.

Bold values denote statistical significant values.

### Non-atrophic duodenal changes and diagnosis

Among children with non-atrophic changes, those who received a diagnosis had more often blood in stools, anemia, and positive celiac serology and had been taking some medication before EGD, whereas the diagnosed and undiagnosed children did not differ in other clinical or laboratory parameters (Table 2). In addition, patients with a diagnosis presented more often with non-specified inflammation and less often with intraepithelial lymphocytosis in the duodenum, more often with any abnormal histological finding in the stomach, and macroscopic and histological findings in colonoscopy, whereas the groups did not differ in esophageal histology or in other histologic or macroscopic duodenal findings (Supplementary Table 1). Of the patients with non-atrophic changes presenting with blood in stools, 9/12 (75%) also underwent colonoscopy at the time of EGD.

**TABLE 2:** Comparison of clinical characteristics at the time of first esophagogastroduodenoscopy (EGD) between children who had non-atrophic duodenal changes and did and did not receive a diagnosis.

	Diagnosis, *n* = 24		No diagnosis, *n* = 27		
		
	*n*	%	*n*	%	*P*-value
Girls	12	50.0	15	55.6	0.692
Symptoms					
* Abdominal pain*	12	50.0	14	51.9	0.895
* Blood in stools*	9	37.5	3	11.1	**0.027**
* Heartburn*	8	33.3	4	14.8	0.120
* Poor growth or weight-loss*	6	25.0	4	14.8	0.485
* Diarrhea*	5	20.8	7	25.9	0.669
* Vomiting*	5	20.8	1	3.7	0.088
* Constipation*	4	16.7	4	14.8	1.000
* Dysphagia*	1	4.2	1	3.7	1.000
Laboratory results					
* Anemia^a^*	12	70.6	4	20.0	**0.002**
* Positive celiac disease serology*^b^**	1	7.7	11	50.0	**0.013**
* Elevated ESR*^c^**	6	46.2	2	12.5	0.092
Previous chronic conditions					
* Asthma or allergy*	8	33.3	5	18.5	0.226
* Autoimmune disease*^d^**	0	0	4	14.8	0.113
Family history of gastrointestinal diseases					
* Celiac disease*	3	12.5	6	22.2	0.473
* Inflammatory bowel disease*	2	8.3	4	14.8	0.473
Use of medication before EGD*^e^*	17	81.0	11	45.8	**0.015**
	**Median**	**Quartiles**	**Median**	**Quartiles**	
	
Age, years	5.5	1.5, 10.9	8.7	4.5, 14.1	0.086
Height, SD*^f^*	1.0	–0.3, 1.0	1.0	–0.2, 1.0	0.667
Body mass index-SDS*^g^*	–0.3	–1.3, 1.1	0.6	0.1, 1.3	0.138

Data were available in >90% of cases except for ^a^72.5, ^b^68.6, ^c^56.9, ^f^68.6, and ^g^56.8%.

^d^Example, celiac disease, type 1 diabetes, rheumatoid arthritis, autoimmune thyroid disease; ^e^no difference in individual drugs, e.g., proton-pump inhibitors, antibiotics, and non-steroidal anti-inflammatory drugs; ESR, erythrocyte sedimentation rate; SD, standard deviation; SDS, standard deviation score. Bold values denote statistical significant values.

### Follow-up

The median follow-up time for all patients with non-atrophic duodenal changes was 10.1 (range 6.1–13.3) years. All 24 children who received an initial diagnosis underwent follow-up, including 12 with a repeat EGD and 10 with a later colonoscopy. Duodenal abnormalities persisted in three cases (chronic non-specific bulbar inflammation and gastric metaplasia, Epstein-Barr virus duodenitis, and tubular adenomas in a patient with familial adenomatous polyposis), whilst nine had normal histology (Supplementary Table 2). Twenty-five (92.6%) of those who did not receive an initial diagnosis underwent a follow-up, during which five out of the 12 children with potential celiac disease developed diagnostic findings, and one patient also ulcerative colitis (Figure 1 and Supplementary Table 2). None of the remaining 13 subjects with negative celiac serology received a later gastrointestinal diagnosis (Figure 1). Five of them underwent a repeat EGD with normal duodenal architecture and 11 received symptomatic treatment with a positive response in six (Supplementary Table 2).

## Discussion

We found non-atrophic duodenal changes to affect 4.4% of children who underwent EGD with systematic sampling. An underlying condition was found in 47.1%, IBD being the most common diagnosis. Receiving a diagnosis was not more likely than among those with normal duodenum, but non-atrophic changes were associated with a higher prevalence of anemia and blood in stools. Altogether, the unexplained duodenal changes appeared to have no major long-term prognostic significance in children with negative celiac serology.

The most frequent non-atrophic duodenal changes here were non-specific mucosal inflammation and intraepithelial lymphocytosis. Singh et al. (19) have reported a prevalence of 7.1% for non-specific duodenal inflammation, 6.1% for eosinophilia, and 0.9% for intraepithelial lymphocytosis in 114 children with an exclusion diagnosis of “functional dyspepsia.” Moreover, Alper et al. (4) reviewed the pathology reports on 2,772 pediatric endoscopies and found a prevalence of 12.7% for any kind of duodenal inflammation, but patients with atrophic changes were also included in this figure. The few other pediatric studies on duodenal findings have focused mainly on intraepithelial lymphocytosis and reported prevalences of 3.0 and 4.3% (12, 13), but it is unclear whether systematic biopsy sampling was performed.

The most common diagnoses other than potential celiac disease in children with non-atrophic duodenal changes were IBD, *H. pylori* infection, and food allergy. The prevalence of diagnoses did not differ between patients with non-atrophic changes and normal mucosa, although the rather small numbers reduce the statistical power. *H. pylori* and food allergy have been linked to duodenal inflammation in earlier studies involving both children and adults (7, 10, 12, 20, 21), whereas the association with IBD is more controversial (12, 22, 23). Other previously reported associations include intestinal bacterial overgrowth, parasitic infections, immunodeficiency, as well as non-steroidal anti-inflammatory drugs, angiotensin receptor blockers, and antibiotics (7, 9, 10, 21, 24). Except for a single case with mevalonate kinase deficiency, none of these were identified here.

Geographic differences and age may affect the prevalence of underlying diagnoses. For instance, *H. pylori* and parasites are less common, and immune-mediated diseases more common in high- than in low-income countries (25–27). Furthermore, immunodeficiencies often present in infancy, celiac disease may appear at any age, and IBD typically manifests in adolescence (7, 10). Another confounder is histopathology, which is seldom pathogenomic and challenging to classify (7, 10, 28). There may also be spatial variation in duodenum (28) and biopsy processing before cutting may have an effect (29). Finally, findings in other parts of the gastrointestinal tract can also be relevant (23, 28, 30), as demonstrated here by the higher frequency of gastric abnormalities in children with IBD. These issues should be considered when generalizing our results to different clinical settings and patient scenarios.

Children with non-atrophic duodenal changes presented more often with blood in stools and anemia than did those without histological abnormalities. Devara et al. (12) reported that children with duodenal lymphocytosis suffer more often from diarrhea and constipation than controls with normal mucosa. In contrast, Kakar et al. (24) did not find differences in clinical presentation between patients with and without duodenitis, but their study also involved adults. The variation in study designs makes it difficult to draw firm conclusions, but the possibility of an underlying organic condition should be remembered in children with unexplained duodenitis and anemia and/or blood in stools, particularly if combined with gastric pathology (7, 12).

Altogether 53% of the children with non-atrophic changes did not receive an initial diagnosis. Five out of the 12 patients with potential celiac disease were diagnosed during the subsequent surveillance, whereas this was the case in none of the seronegative patients. Devara et al. (12) recently reported a histological recovery rate of 83% in pediatric lymphocytic duodenosis, median of 3.8 (range 0–191) months after EGD. In line with us, children who later received a diagnosis had laboratory markers indicating celiac disease or IBD (12). Lähdeaho et al. (14) retrospectively followed the development of celiac disease in 76 children with intraepithelial lymphocytosis and observed five new diagnoses within 8–28 years. Taken together, seronegative children with non-atrophic duodenal changes but no identifiable underlying disease appear to have a favorable long-term prognosis.

The main strengths of the present study were the large cohort of consecutive children who had undergone systematic duodenal sampling and the availability of long-term follow-up data. The main limitation was the retrospective design, predisposing to biased data on clinical and laboratory parameters. Furthermore, duodenal histology was not systemically double-reviewed, and bulb samples were available from less than half of the patients. It must also be emphasized that, due to the high threshold for invasive procedures in children, repeat endoscopies were conducted only on a subgroup of subjects with non-atrophic changes.

## Conclusion

Although non-atrophic duodenal changes are a relatively common finding in pediatric EGD, with the exception of potential celiac disease, they do not appear to have major prognostic significance. However, the presence of an underlying organic condition should be kept in mind, particularly in children presenting with blood in stools or anemia and histological abnormalities in other parts of the gastrointestinal tract.

## Data availability statement

The original contributions presented in this study are included in the article/Supplementary material, further inquiries can be directed to the corresponding author.

## Ethics statement

Ethical review and approval was not required for the study on human participants in accordance with the local legislation and institutional requirements. Written informed consent from the participants or their legal guardian/next of kin was not required to participate in this study in accordance with the national legislation and the institutional requirements.

## Author contributions

LK and KK designed and supervised the study. SK, MR, PH, and MV collected the data. SK and HH were responsible for statistical analyses. SK, LK, and KK drafted the manuscript and wrote the final version. All authors interpreted the results, approved the final draft submitted, and agreed to be accountable for all aspects of the work.

## Abbreviations

BMI: body mass index
CD: celiac disease
EGD: esophagogastroduodenoscopy
EMA: IgA-class endomysial antibodies
ESR: erythrocyte sedimentation rate
*H. pylori*: *Helicobacter pylori*
Hb: hemoglobin
IBD: inflammatory bowel disease
SD: standard deviation
SDS: standard deviation score
TGA: IgA-class tissue transglutaminase antibodies.
