# Supplementary material for: Differential diagnosis and long-term outcomes of non-atrophic duodenal changes in children
Source: Front Pediatr. 2022 Aug 29;10:982623. doi: 10.3389/fped.2022.982623 (PMC9464825; doi:10.3389/fped.2022.982623)
Supplement: Supplementary file 1 [file Table_1.DOCX]

| **Supplementary table 1.** Comparison of macroscopic and histologic findings at the time of first esophagogastroduodenoscopy between children who had non-atrophic duodenal changes and did and did not receive a diagnosis | | | | | | | | | | |
| --- | --- | --- | --- | --- | --- | --- | --- | --- | --- | --- |
|  | |  | **Diagnosis**  **n=24** | |  |  | **No diagnosis**  **n=27** | |  |  |
|  | | n | | % | | n | | % | | P value |
| Macroscopic findings in duodenum | | 18 | | 75.0 | | 18 | | 66.7 | | 0.514 |
| Histologic findings in duodenum | |  | |  | |  | |  | |  |
|  | *Non-specific inflammation* | 13 | | 54.2 | | 6 | | 22.2 | | **0.019** |
|  | *Intraepithelial lymphocytosis* | 3 | | 12.5 | | 11 | | 40.7 | | **0.024** |
|  | *Marked lymphoid hyperplasia* | 2 | | 8.3 | | 2 | | 7.4 | | 1.000 |
|  | *Mast cell infiltration* | 2 | | 8.3 | | 1 | | 3.7 | | 0.483 |
|  | *Lymphangiectasia* | 1 | | 4.2 | | 4 | | 7.4 | | 0.354 |
|  | *Other ^a^* | 7 | | 29.1 | | 4 | | 14.8 | | 0.214 |
| Duodenal bulb sample taken | | 10 | | 41.7 | | 11 | | 40.7 | | 0.947 |
|  | *Histologic findings present* | 7 | | 70.0 | | 10 | | 90.9 | | 0.624 |
| Histologic findings in ventricle | | 14 | | 58.3 | | 5 | | 18.5 | | **0.003** |
| Histologic findings in esophagus | | 7 | | 31.8 | | 4 | | 15.4 | | 0.117 |
| Colonoscopy performed | | 14 | | 58.3 | | 8 | | 29.6 | | **0.039** |
|  | *Macroscopic findings present* | 10 | | 71.4 | | 1 | | 12.5 | | **0.024** |
|  | *Histologic findings present* | 11 | | 78.6 | | 2 | | 25.0 | | **0.026** |
| ^a^ Gastric metaplasia (n=3), tubular adenoma (n=1), abnormal villous shape (n=1), bulbar regeneration (n=1), eosinophilic infiltrate (n=1), congestion (n=1), Epstein-Barr infection (n=1), ulcer (n=1), mucosal hematoma (n=1). | | | | | | | | | | |
