# Supplementary material for: Differential diagnosis and long-term outcomes of non-atrophic duodenal changes in children
Source: Front Pediatr. 2022 Aug 29;10:982623. doi: 10.3389/fped.2022.982623 (PMC9464825; doi:10.3389/fped.2022.982623)
Supplement: Supplementary file 2 [file Table_2.DOCX]

| **Supplementary table 2.** Long-term outcomes in children who had non-atrophic duodenal changes and did and did not receive a diagnosis at the time of first esophagogastroduodenoscopy | | | | | | | | | | |
| --- | --- | --- | --- | --- | --- | --- | --- | --- | --- | --- |
|  | | |  | **Diagnosis, n=24** | |  |  | **No diagnosis, n=27** | |  |
|  | | | n | | % | | n | | % | |
| Follow-up implemented | | | 24 | | 100 | | 25 | | 92.6 | |
|  | *Follow-up elsewhere/lacking data* | | 13 | | 54.2 | | 4 | | 16.0^a^ | |
|  | *Returned at some point* | | 6 | | 46.2^b^ | | 1 | | 25.0^c^ | |
| Mainly pharmaceutical treatment | | | 22^d^ | | 91.7 | | 12^e^ | | 44.4 | |
| Mainly elimination diet | | | 3 | | 12.5 | | 4^f^ | | 14.8 | |
| Positive treatment response | | | 16 | | 66.7 | | 10 | | 62.5^g^ | |
| New esophagogastroduodenoscopy | | | 12 | | 50.0 | | 8 | | 29.6 | |
|  | | *Duodenal abnormalities* | 3^h^ | | 25.0 | | 3^i^ | | 37.5 | |
| New colonoscopy | | | 10 | | 41.7 | | 4 | | 14.8 | |
| Imaging studies in follow-up ^j^ | | | 6 | | 25.0 | | 7 | | 25.9 | |
| ^d^ E.g., proton-pump inhibitor, prednisolone, mesalazine and iron supplement, two subjects also had an elimination diet,  ^e^ e.g., proton-pump inhibitor, osmotic laxative, or iron supplement, two patients also had an elimination diet; ^f^ milk, wheat or histamine releasing foods, all four had also pharmaceutical treatments; ^h^ non-specific bulbar inflammation and gastric metaplasia, Epstein-Barr virus duodenitis, tubular adenomas (patient with familial adenomatous polyposis, FAP). In further EGD’s all abnormalities had disappeared except for those associated with FAP; ^i^ Partial, subtotal and total villous atrophy, all three received a celiac disease diagnosis; ^j^ abdominal ultrasonography, magnetic resonance enterography, capsule endoscopy; esophageal pH monitoring; ^a^4/25, ^b^6/13, ^c^1/4, ^g^10/16. | | | | | | | | | | |
